# Supplementary material for: Green Synthesis of Endolichenic Fungi Functionalized Silver Nanoparticles: The Role in Antimicrobial, Anti-Cancer, and Mosquitocidal Activities
Source: Int J Mol Sci. 2022 Sep 13;23(18):10626. doi: 10.3390/ijms231810626 (PMC9502095; doi:10.3390/ijms231810626)
Supplement: Supplementary file 1 [file ijms-23-10626-s001.zip › ijms-1851154-supplementary.pdf]

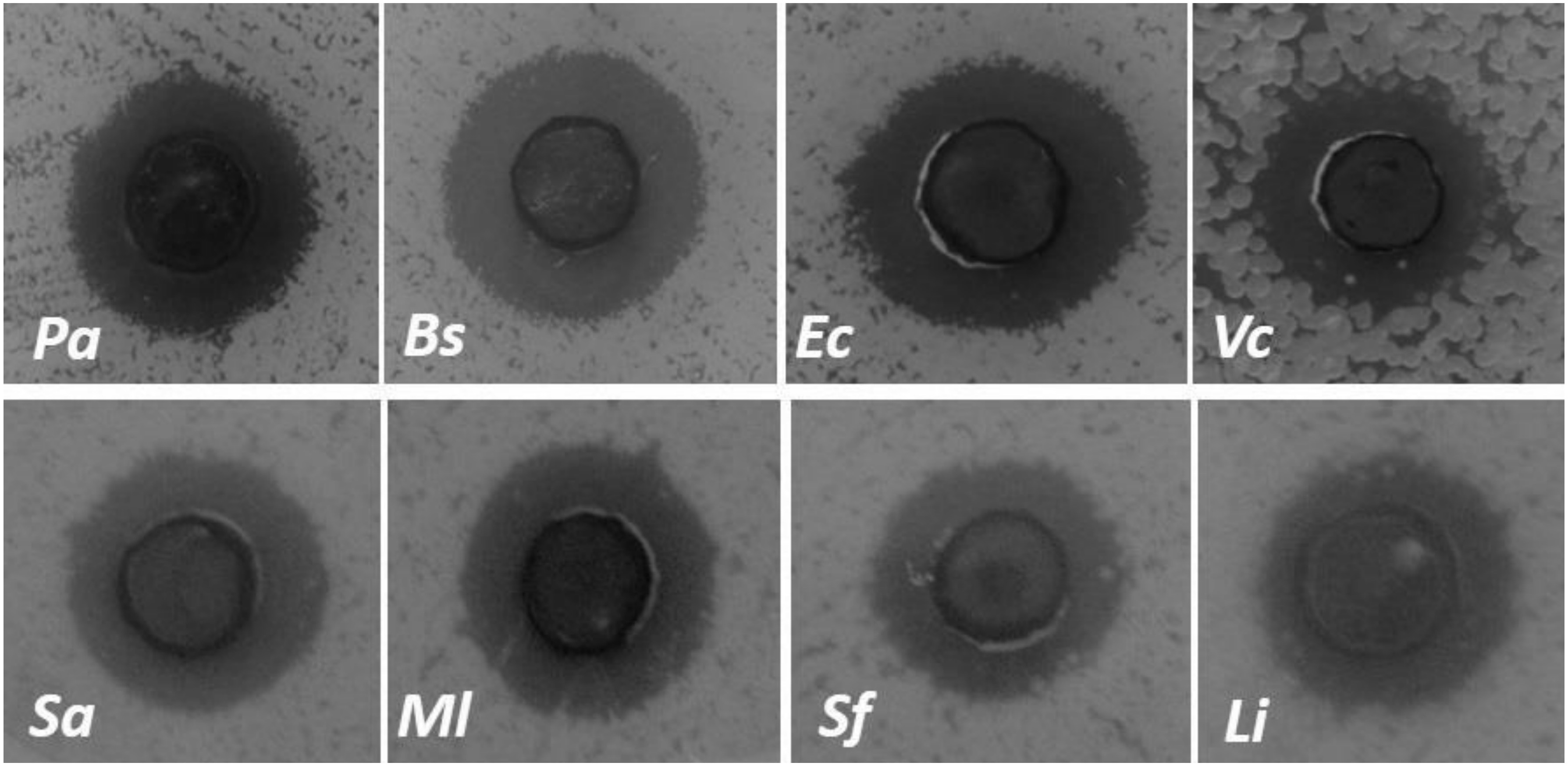

Supplementary Figure S1: Antimicrobial activity (ZI) of *T. funiculosus* –AgNPs by agar well diffusion method: ***Pa*****:** *Pseudomonas aeruginosa*; ***Bs***: *Bacillus subtulis*; ***Ec***: *Escherichia coli*; ***Vc:*** *Vibrio cholera*; ***Sa***: *Staphylococcus aureus*; ***Ml***: *Micrococcus luteus*; ***Sf***: *Streptococcus faecalis;* ***Li*****:** *Listeria innocua.*

Supplementary Table-S1:

Test Microorganisms	% of inhibition ±S.D. (µg/mL)											MIC IC_{50} (µg/mL)
	0 µg/mL	10 µg/mL	20 µg/mL	30 µg/mL	40 µg/mL	50 µg/mL	60 µg/mL	70 µg/mL	80 µg/mL	90 µg/mL	100 µg/mL	
Staphylococcus aureus	0.00	0.00	2.27± 0.12	31.07± 0.74	40.67± 0.45	50.77± 0.63	81.00± 1.41	99.00± 0.62	99.57± 1.23	99.07± 0.74	100.00± 0.82	43.94±0.29
Listeria innocua	0.00	0.00	13.93± 1.41	23.53± 0.68	28.20± 0.67	35.17± 0.94	43.07± 1.38	50.40± 0.43	62.13± 0.87	91.33± 1.43	99.80± 1.07	68.94±0.30
Micrococcus luteus	0.00	12.80± 1.53	23.17± 0.70	30.57± 0.49	41.47± 0.46	52.23± 0.90	72.40± 1.35	83.53± 1.20	99.37± 0.34	99.93± 0.17	99.97± 0.86	45.51±0.21
Streptococcus faecalis	0.00	0.00	0.00	21.90± 1.42	35.27± 1.31	45.70± 0.29	63.17± 1.39	72.57± 1.77	90.63± 0.29	99.07± 0.74	100.00± 0.82	52.54±0.16
Vibrio cholerae	0.00	0.00	2.73± 0.46	4.13± 0.17	7.73± 0.96	17.50± 1.80	41.67± 1.03	50.97±0.76	81.70± 2.09	99.00± 0.62	99.57± 1.23	63.69±0.25
Escherichia coli	0.00	3.40± 0.08	6.40± 0.16	15.50± 0.22	40.67± 0.45	50.77± 0.63	81.00± 1.41	99.00± 0.62	99.57± 1.23	100.00± 0.62	100.00± 0.71	43.94±0.29
Pseudomonas aeruginosa	0.00	20.27± 0.31	43.33± 2.21	50.77± 0.63	82.67± 1.70	99.00± 0.62	99.57± 1.23	100.00± 0.00	100.00± 0.00	-	-	21.68±0.72
Bacillus subtilis	0.00	0.00	15.50± 0.22	41.13± 1.46	50.77± 0.63	81.17± 1.40	99.23± 0.86	100.00± 0.59	-	-	-	33.60±0.61
